# New psychometric evidence from the Revised Mental Health Inventory (R-MHI-5) in Peruvian adolescents from a network psychometrics approach

**DOI:** 10.1186/s40359-024-01543-w

**Published:** 2024-01-29

**Authors:** Estefany Rojas-Mendoza, Vaneryn Alania-Marin, Aaron Travezaño-Cabrera

**Affiliations:** Escuela de psicología, Universidad Peruana Unión, Lima, Peru

**Keywords:** Mental health, Confirmatory factor analysis, Exploratory graph analysis, Peru, Adolescents

## Abstract

**Background:**

Mental health is an aspect that has been relegated in recent years, prioritizing physical health care. However, there are more and more mental problems and disorders worldwide. In this context, adolescents are considered at risk for developing psychological difficulties due to the important transition stage they are going through. Given this, an adequate measurement of mental health in the adolescent population is necessary. This research aims to evaluate the internal structure and estimate the reliability of the R-MHI-5.

**Method:**

The sample covered 662 adolescents (55.2% men and 44.7% women) aged 12 to 17 years (M = 14.5; SD = 1.6).

**Results:**

Exploratory graph analysis revealed a two-dimensional structure encompassing well-being and psychological distress. Furthermore, confirmatory factor analysis results indicated that a model with two related factors demonstrated superior fit indices (CFI = 0.99; TLI = 0.99; SRMR = 0.04; RMSEA = 0.101) in comparison to a one-dimensional model (CFI = 0.85; TLI = 0.71; SRMR = 0.23; RMSEA = 0.451). Additionally, adequate values were found in the reliability of the dimensions.

**Conclusion:**

It is concluded that the R-MHI-5 is an instrument with robust psychometric evidence from the perspective of the EGA and CFA, providing adequate evidence of reliability and validity so that it can be used effectively in future research and prevention and intervention processes. Furthermore, our study is the first to provide psychometric evidence of the scale from a media approach in Peruvian adolescents.

## Introduction

Mental health depends mainly on external and internal factors that play a protective or risky role in the well-being of people in their different stages of development [1]. In this sense, adolescents are considered one of the most vulnerable populations since they are in a crucial development period [2]. This vulnerability has increased in the context of the pandemic, bringing negative consequences for their physical and mental health [3]. International studies found that there is an increase in levels of anxiety and depression in adolescents [3–6], symptoms that have generated a greater presence of somatic problems, manifestations of anger and irritability, and behavioral problems, being potential risk factors for their mental health [3, 7, 8] in this regard, the World Health Organization (WHO) [9] reported that one in seven adolescents between the ages of 10 and 17 have a psychological disorder.

Regarding Latin America and the Caribbean, the United Nations Children's Fund(UNICEF) [10], in September and February 2021, measured 8,444 adolescents and young people, whose ages range from 13 to 29, to investigate the impact of mental health due to COVID-19. Among the participants, an important group showed symptoms of anxiety (27%) and depression (15%); 46% indicated a decrease in motivation to carry out activities they previously enjoyed, and 36% felt less motivated to carry out their usual tasks. In addition, according to estimates from the Ministry of Health [11], 30% of adolescents in Peru have a high probability of developing behavioral, emotional, or attentional mental health difficulties. In comparison, 9% of adolescents have suffered from depression at some point, and one in eight is predisposed to developing a mental pathology during his life trajectory [12].

All of the above indicates the detrimental impact on adolescents’ mental health. The WHO defines the latter [13] as “a state of complete physical, mental, and social well-being and not simply the absence of disease” (p. 1). Likewise, the Pan American Health Organization [14] mentions that mental health goes far beyond the lack of mental illness but constitutes the basis for the well-being and effective functioning of the individual. Initially, mental health was conceptualized under a one-dimensional model, where the absence of psychopathological symptoms was a positive indicator of mental health [15]. However, currently, mental health includes a comprehensive evaluation of the human being, where positive indicators of well-being are integrated into the main aspects: physical, mental, and social. The Dual Factor Mental Health Model (MDSM) is developed in this context [15], which affirms that the presence of well-being and the absence of psychopathological symptoms are not opposite poles within a single dimension but that they constitute two different factors of mental health, which in turn are negatively related [16]. The evaluation of these two aspects provides a comprehensive understanding and evaluation of mental health by evaluating well-being and psychological distress [17–20].

Within this contextual framework, studies in the adolescent population found that mental health is associated with suicidal ideation [21], difficulties in emotional regulation and its relationship with aggression [22], stress [23], affectivity, and quality of life [24]. In addition, studies reported that the absence of family communication [25], domestic violence [26], and inappropriate parenting practices [27] negatively influence mental health. On the other hand, other research found that mental health has a link with self-esteem [28], self-concept [29], self-efficacy [30], coping styles, focused on problem-solving and seeking social support [31], hope in God [32] and religiosity [33].

According to the MDSM, it is essential to have instruments that evaluate negative and positive aspects of mental health [15].. An instrument created under this approach is the Mental Health Inventory (MHI), comprised of 38 items that evaluate well-being and psychological distress [34]. Subsequently, an abbreviated version was created, made up of five items, called MHI-5 [35]. This instrument has the same effectiveness as the extended version and is quick to apply compared to other instruments [36–45].

On the other hand, given its ease of response and rapid evaluation, as well as its evidence of validity and reliability, it is considered an adequate instrument, also allowing the detection and diagnosis in the general population of anxiety, depression, and panic disorders [46–48]. Concerning this, various studies applied the MHI-5 in different groups and cultures, such as Portugal [42], American women [49], Brazil [50], Saudi Arabian college students [51], Dutch population [52], Spanish children and adolescents [53], Australia [54] and Finland [55] among others.

Regarding the psychometric properties of the MHI-5, there is little research on adolescents; in this regard, a study carried out on Portuguese adolescents reported a single factor for the scale with an good reliability index (α = 0.82) [42]. In addition to this, an investigation in Spanish adolescents (10 to 15 years old) found a new factorial structure that includes well-being and anguish, and that explains 69.2% of the total variance, reporting an adequate total internal consistency (α = 0.71) and for the well-being dimensions (α = 0.70) and psychological distress (α = 0.71) [53]. Likewise, it presents a reduced response format of four alternatives and not the six proposed, this version was referred to as R-MHI-5 [35].

In addition, in Latin America, few studies have been found that report psychometric evidence of the R-MHI-5 in adolescents. In this regard, a study in Peru of secondary school adolescents reported the existence of two factors, with acceptable adjustment indices (CFI = 0.90; RMSEA = 0.12; SRMR = 0.18) and adequate reliability (α = 0.70) [56]. In comparison, a recent investigation in young Peruvian university students reported adequate fit indices for the model with two related factors (CFI = 0.99; TLI = 0.99; RMSEA = 0.071) as well as good reliability for the well-being dimension (ω = 0.75) and psychological distress (ω = 0.79) [57].

In relation to previous studies, they exhibit certain limitations, particularly in the study involving Peruvian adolescents, where a restricted number of participants were involved. This might lead to a suboptimal implementation of CFA, given that a larger sample size is requisite for this analysis [56]. In addition, the studies show a variation in terms of the structure of the R-MHI-5. Therefore, more evidence is still needed to explore the structure of the scale, using alternative statistical methods for this purpose. In this context, Exploratory Graph Analysis (EGA) is a suitable technique for estimating the number of dimensions [58]. One of the advantages of the EGA, in the first place, is that it allows straightforward interpretation of the elements (items) that belong to each dimension through a visual scheme without having to interpret a factorial matrix. Secondly, it allows observing the associations between elements and dimensions, identifying the key variables and their function within the graph. Third, EGA requires fewer steps to estimate dimensionality, and its analysis is not affected by the sum of items, the components of the instrument, or the sample size [58–60]. Therefore, EGA is an adequate and reliable tool to explore the factorial structure and the interactions between the dimensions of mental health measured by the R-MHI-5.

Therefore, due to the absence of psychometric investigations of the R-MHI-5 in Peru and the differences in its internal structure that previous investigations have reported, this research aims to evaluate the internal structure and estimate the reliability of the R-MHI-5.

## Method

### Participants

The sample selection was carried out using non-probabilistic sampling for convenience, a method that allows the choice of study elements based on the researcher’s criteria, depending on the easy accessibility and closeness it has with the participants. Thus, the following inclusion criteria were taken into account: (a) have the consent of the parents, (b) voluntary participation in the research, (c) be studying a level of secondary education or higher education, (d) ages 12 to 17, (e) study in an institution belonging to Lima East. Likewise, the following exclusion criteria were used: (a) not completing all the questionnaires, (b) randomly answering the questionnaires. We worked with a sample of 662 adolescents of both sexes (55.2% men and 44.7% women) between 12 and 17 years of age (M = 14.5; SD = 1.6). The adolescents are from Peru; most come from the coast (78.0%), 17.5% from the mountain, and 4.3% from the jungle. A large part of the population (58.9%) lives with their parents and siblings, while 24.7% live only with their mother and siblings. Regarding the study center, 70.9% study in a public institution, and 29.0% do so in a private institution. Finally, a significant number of adolescents (29.3%) have ever received psychological treatment, while 70.6% of participants do not receive or have received treatment. Details of the sociodemographic data are specified in Table 1.

**Table 1:** Sociodemographic data

Variables	n	%
**Sex**		
Male	366	55.2%
Female	296	44.7%
**Age**		
12–14	290	43.8%
15–17	372	56.1%
**Educational institution**		
Public	470	70.9%
Private	192	29.0%
**Origin**		
Coast	517	78.0%
Mountain	116	17.5%
Jungle	29	4.3%
**Religion**		
Catholic	266	40.1%
Evangelical	72	10.8%
Adventist	143	21.6%
Other	181	27.3%
**Parents’ marital status**		
Married	255	38.5%
Cohabitants	215	32.4%
Separated/divorced	192	29.0%
**Who do you live with?**		
Dad and mom	51	7.7%
Dad, mom, and siblings	390	58.9%
Only mom and siblings	164	24.7%
Only dad and siblings	19	2.8%
Only mom	18	2.7%
Only dad	13	1.9%
With other relatives or guardians	7	1.0%
**Receive or have received psychological treatment**		
Yes	194	29.3%
No	468	70.6%

### Instrument

#### Revised Mental Health Inventory-5 (R-MHI-5)

Berwick created the Mental Health Inventory [35], adapted to Spanish by Rivera-Riquelme et al. [53] and validated in the Peruvian context by Vilca et al. [57]. The R-MHI-5 is an instrument to evaluate mental health in adolescents and adults. The inventory is made up of 2 dimensions; one of them evaluates the presence of psychological well-being (items 2 and 4), while the other estimates the absence of psychological distress through inverse items (items 1, 3, and 5), both respond to the state of the person’s mood during the last month. It is made up of 5 items, with response alternatives on a Likert scale ranging from never (0), sometimes [1], often [2] and always [3]. In relation to its psychometric properties, the scale showed validity based on the internal structure in the unidimensional model (CFI = 0.99; TLI = 0.99; RMSEA = 0.071). It also presents adequate levels of internal consistency for the dimensions well-being (ω = 0.75) and psychological distress (ω = 0.79).

### Procedure

A virtual and physical form was used, made up of three sections. In the first section, the informed consent addressed to the parents and the permission to the students were considered; in the second, the sociodemographic information, and the third covered the items of the instruments to be evaluated. WhatsApp sent the form link. Similarly, data collection took place in person after obtaining institutional permission, which was secured through the submission of a request letter issued by Universidad Peruana Unión and signed by the director of the School of Psychology. The validation of the letter was approved by the leader of the educational institutions. Subsequently, parental approval for the participation of their minor children was obtained. Finally, each student who received informed consent from their parents and provided assent to participate in the study was administered the inventory. The data collection period commenced on August 29 and concluded on September 30, 2022.

### Statistical analysis

First, descriptive statistics such as skewness and kurtosis, standard deviation, and mean were analyzed. To conduct Exploratory Graph Analysis (EGA), a Gaussian Graphical Model (GGM) was employed, estimated using the graphical least absolute shrinkage and selection operator (GLASSO) [61].The Walktrap algorithm [62] was utilized to determine the number of factors. Concerning network loadings, small (0.15), moderate (0.25), and large (0.35) loading values were considered [63]. Additionally, the exploratory graph analysis bootstrap approach (bootEGA) with 1000 replications was employed to determine the structural consistency and stability of the items, with values exceeding 0.75 considered acceptable [64].

Then, a confirmatory factor analysis (CFA) was performed using the weighted least squares mean and variance adjusted (WLSMV) to evaluate that the model presents good fit indices. Among the indices considered are the CFI and TLI, where it is expected to get values greater than 0.95 [65]. Also, the indices of SRMR and RMSEA must present values equal to or less than 0.08 [66].

The omega coefficient [67] was used to estimate the internal consistency, where a value > 0.80 is adequate [68]. The analysis of the described data was developed using the R program [69] and the RStudio graphic interface [70].

### Ethical aspects

This study was evaluated and approved by the Universidad Peruana Unión Ethics Committee under the number 2022-CE-FCS-UPeU-088 and with the project title “New psychometric evidence from the Mental Health Inventory (R-MHI-5) in Peruvian adolescents from a network psychometrics approach”. Similarly, the present study was adjusted to what was indicated by the Declaration of Helsinki [71] to respect the ethical principles involved in human research. In this sense, the principle of autonomy was taken into account by granting informed consent to the participants before data collection to ensure that their participation in the research was of their own free will and responsibility for themselves. In addition, the principle of justice was complied with since the study did not cause any harm to the participants. However, if what was requested in the survey produced any affectation on an emotional level, a contact number was provided through which emotional support was provided as well. Finally, the principle of confidentiality was respected since the information of the institution and the participants involved in the study was protected.

## Results

### Descriptive analysis

Table 2 shows that item 4 (‘During the last month, how often have you felt happy?’) presents the highest mean score in the total sample (M = 1.76). On the other hand, item 3 is shown as a low score (‘During the last month, how often have you felt discouraged or sad?’) (M = 1.33). Likewise, it can be shown that the items showed adequate indices of asymmetry and kurtosis (± 1.5) in the total sample.

**Table 2:** Descriptive analysis of the items

Items	Total sample (*n* = 662)			
	M	SD	g1	g2
M1	1.50	0.76	0.03	− 0.35
M2	1.71	0.94	0.00	− 1.06
M3	1.33	0.93	0.13	− 0.87
M4	1.76	0.87	− 0.01	− 0.92
M5	1.68	0.88	0.15	− 0.96

### Exploratory graph analysis

**Fig. 1:**
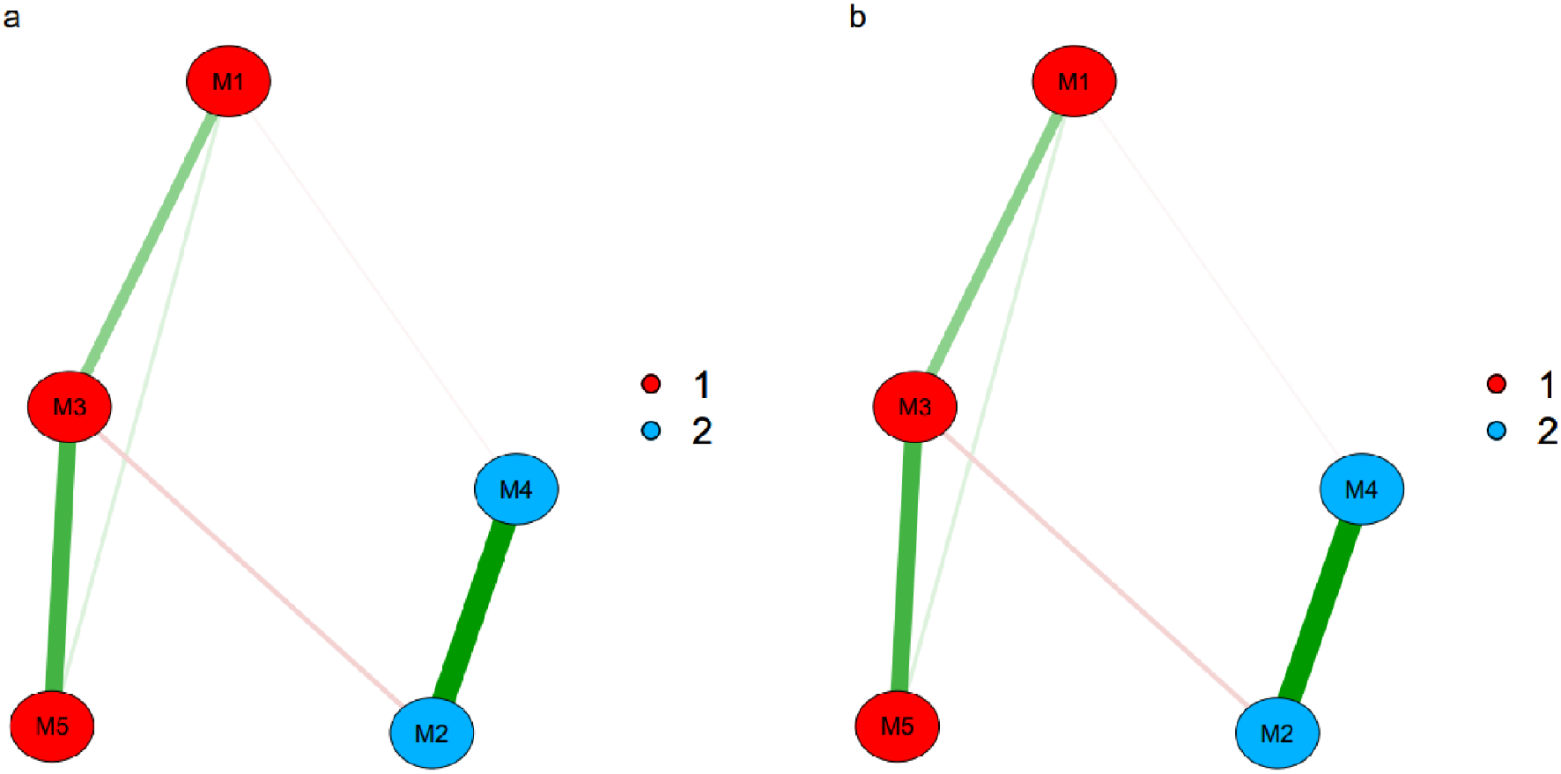
Dimensionality assessed via EGA (**a**) and bootEGA (**b**) for the R-MHI-5

Figure 1 shows the estimated dimensionality of the R-MHI-5 using the EGA, where two dimensions were found (well-being and psychological distress). Regarding the network loadings, the dimension of psychological distress presents adequate values in items 1 (0.26), 3 (0.59), and 5 (0.39). In contrast, items 2 and 4, belonging to the psychological well-being dimension, obtained values of 0.49. In addition, after 1000 bootstrap replications, the results show that the elements had identical locations in both medias.

**Fig. 2:**
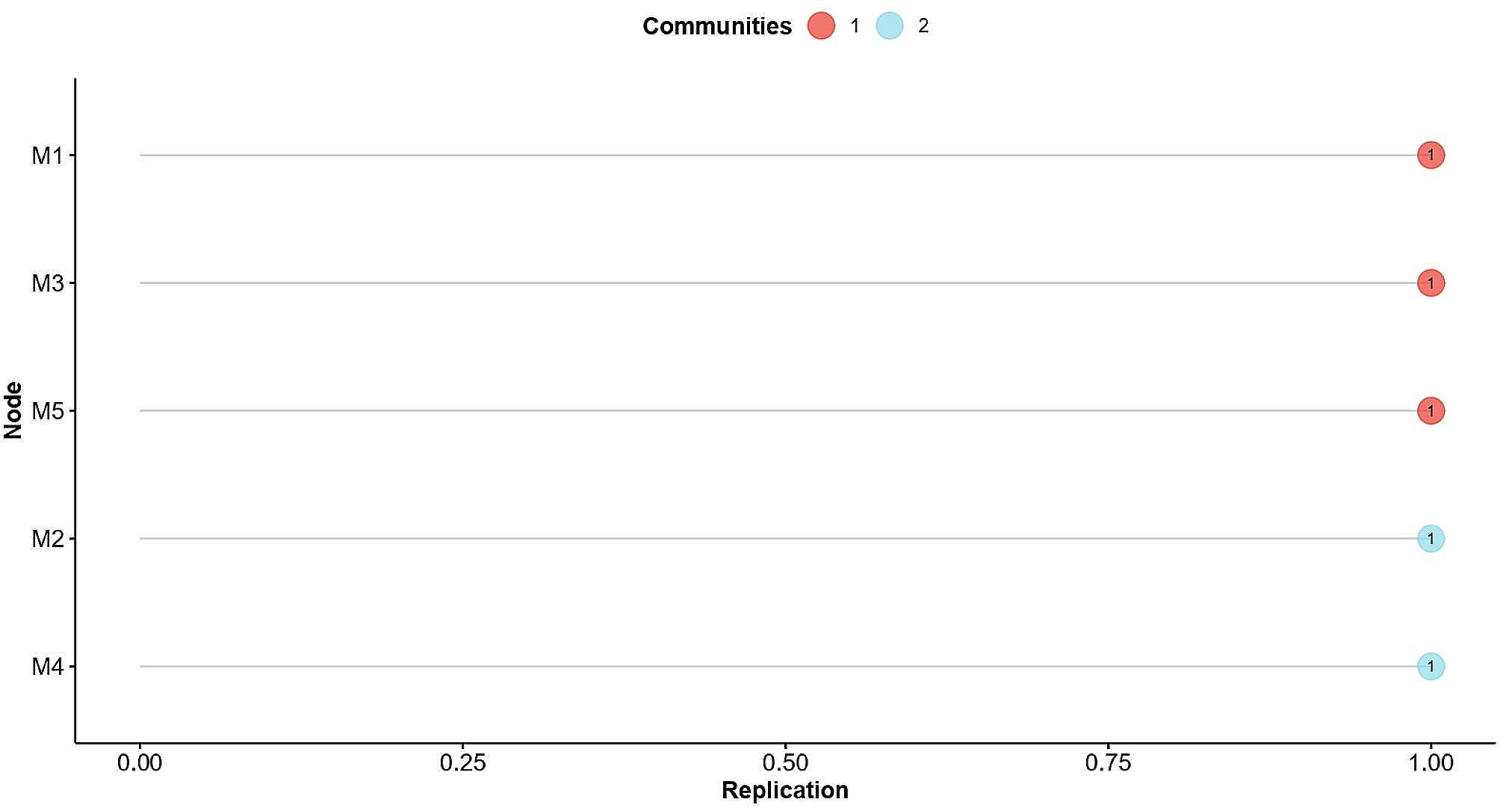
Stability of the R-MHI-5 items

In Fig. 2, the stability of the items was evaluated to inspect the proportion of times that each item is replicated within each dimension; the results indicate that all the items were consistently identified, replicating well (100%) within their empirically designated dimension by EGA. Regarding the structural consistency, it was found that the two-dimensional structure was replicated 100% of the time.

### Confirmatory factor analysis

**Fig. 3:**
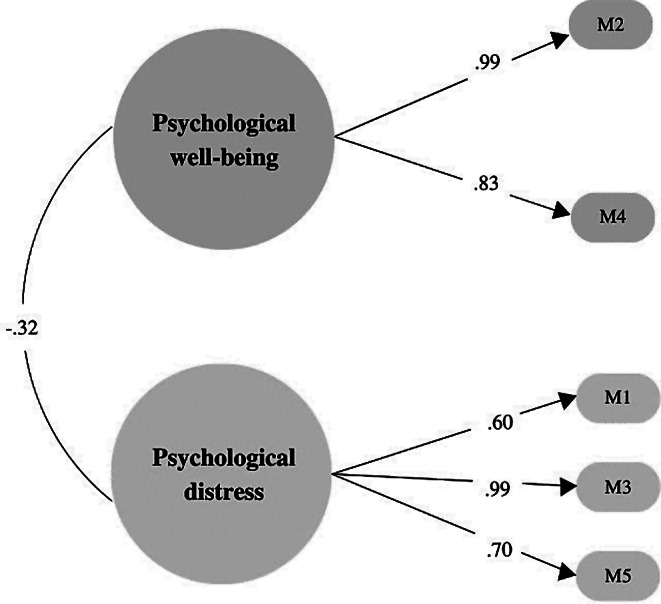
Two-factor correlated model

Table 3 shows the results of the first one-dimensional model put to the test, which did not have adequate fit indices (χ2 = 678.70; df = 5; *p* =.000; CFI = 0.85; TLI = 0.71; SRMR = 0.23; RMSEA = 0.451 [IC 90% 0.423 − 0.480]). Subsequently, a structural model with two related factors was tested, which yielded adequate fit indices (χ2 = 30.99; df = 4; *p* =.000; CFI = 0.99; TLI = 0.99; SRMR = 0.04; RMSEA = 0.101 [IC 90% 0.070 − 0.136]). Regarding the relationship between both factors, they show a negative and acceptable relationship (− 0.32) (see Fig. 3).

**Table 3:** Two-dimensional model fit indices

Models	X^2^	df	p	RMSEA	IC 90%	SRMR	CFI	TLI
One-dimensional model	678.70	5	0.000	0.451	0.423– 0.480	0.225	0.85	0.71
Two related factors model	30.99	4	0.000	0.101	0.070–0.136	0.043	0.99	0.99

Figure 3 shows that the structural model is adequately represented by its items since they all present relatively high factor loads. It is necessary to mention that item 2 (‘During the last month, how often have you felt calm and at peace?’) and item 3 (‘In the previous month, how often have you felt discouraged or sad?’) are the ones that have higher factorial weight (0.99); therefore, they are the items that best represent well-being and psychological distress, respectively.

### Scale reliability

The results obtained from the total sample report that the dimensions of psychological well-being (ω = 0.88) and psychological distress (ω = 0.79) present adequate reliability indices.

## Discussion

Adolescence is considered one of the most vulnerable populations since they are in a crucial development period [2]. This has increased in the context of the health emergency that the country faced, causing negative repercussions on physical and mental health [3]. In this regard, it is crucial to have a valid and reliable tool for detecting and preventing certain mental disorders in the study population. For this reason, this research aimed to estimate the psychometric properties of the R-MHI-5 in Peruvian adolescents.

Concerning the results reported by EGA, an internal structure composed of two dimensions is confirmed: well-being and psychological distress. This study is the first to use EGA as a psychometric exploration method for the R-MHI-5. The advantage of the EGA, in the first place, is that it allows one to quickly interpret the number of dimensions and which elements (items) belong to each dimension through a graphical representation, without the need to interpret a factorial load matrix [72]. Second, the media model provides a new alternative to estimate the dimensionality of mental health since the approach suggests that psychological variables connect and reinforce each other, forming a causally connected system without necessarily detecting a common latent variable [73]. Therefore, within the network models, mental health is considered a system that arises from interacting indicators, forming the construct that implies that the variables (items) are not necessarily causes of mental health, as usually represented by factorial models. In other words, mental health might not be a direct causal factor of life satisfaction and well-being, but rather, mental health might arise due to the mutual interaction between these elements [74].

Regarding internal validity, the results confirm a two-dimensional model with appropriate well-being and psychological distress adjustment indices. This reaffirms the existence of a model of two correlated factors over a one-dimensional model; therefore, it is decided to choose this model. Likewise, the results are similar to those reported by previous studies, such as the one conducted in Spain with an adolescent sample, which reported a bidimensional structure [53]. Similarly, a study in Peruvian high school students identified a two-factor model [56]. In the same context, there is recent research in university students that reported adequate fit indices for a model with two related factors [57]. Moreover, it empirically supports the theoretical proposal of the MDSM, which considers that well-being and the absence of psychopathological symptoms are not opposite poles within the same dimension but that they constitute two different factors of mental health, negatively associated [16]. The evaluation of both aspects provides a comprehensive understanding and evaluation of mental health [19, 20].

On the other hand, it is important to indicate that, according to the results, a high RMSEA was obtained. Simulation studies have shown that the degrees of freedom could influence the RMSEA value; if the results present low degrees of freedom, this can influence the RMSEA, resulting in high values [75, 76]. In this sense, this study had a value of 4 in terms of degrees of freedom, which explains the RMSEA values obtained (0.101). Given the inherent limitations of RMSEA, support has been provided for the utilization of alternative fit indices, such as SRMR and CFI, due to their lower susceptibility to influences on their values when the model features a limited number of degrees of freedom [77]. It is noteworthy that, in the study, the values of these fit indices were deemed appropriate.

Concerning the results reported by EGA, an internal structure composed of two dimensions is confirmed: well-being and psychological distress. This study is the first to use EGA as a psychometric exploration method for the R-MHI-5. The advantage of the EGA, in the first place, is that it allows one to quickly interpret the number of dimensions and which elements (items) belong to each dimension through a graphical representation, without the need to interpret a factorial load matrix [72]. Second, the media model provides a new alternative to estimate the dimensionality of mental health since the approach suggests that psychological variables connect and reinforce each other, forming a causally connected system without necessarily detecting a common latent variable [73]. Therefore, within the network models, mental health is considered a system that arises from interacting indicators, forming the construct that implies that the variables (items) are not necessarily causes of mental health, as usually represented by factorial models. In other words, mental health might not be a direct causal factor of life satisfaction and well-being, but rather, mental health might arise due to the mutual interaction between these elements [74].

Finally, in terms of reliability, adequate values were obtained, which are similar to the results obtained by previous investigations [53, 56, 57]. It is necessary to specify that the omega confers a significant benefit by yielding more precise reliability values [78]. When performing analyses involving factor loadings, this coefficient facilitates obtaining more consistent results and accurately represents the underlying level of reliability, regardless of the number of items used [79].

Within the limitations, in the first instance, it is important to point out the type of sampling carried out, which was intentionally non-probabilistic, which is a limitation to generalizing the results. Secondly, not including other measurement scales prevents knowing the validity of the R-MHI-5 concerning other constructs. Third, not making the invariance based on the particular characteristics of the participants limits the support for conducting comparative studies. Fourth, the test-retest reliability was not verified, which makes it challenging to demonstrate the temporal stability of the instrument. Finally, the study utilized the R-MHI-5 version and identified two dimensions; however, it is recommended that future research explores the extended version, MHI-36, using EGA. Despite the limitations, the scientific contribution made in this study is highlighted since it is the first in Latin America to demonstrate new psychometric evidence of the R-MHI-5 from an EGA.

In short, the results show that the R-MHI-5 is supposed to be a valid, brief, and reliable instrument for measuring mental health in adolescents, with adequate psychometric evidence based on EGA and CFA. In addition, this scientific contribution is expected to fill the gap in the measurement and research of mental health and motivate more psychometric studies that use both traditional and current methods. One is the EGA, a model that has shown greater precision and efficiency than other analyses traditionally used to evaluate the internal structure. Finally, this evidence will help private and public entities to use the R-MHI-5 for the detection, screening, and measurement of health gaps in the adolescent population to take action to carry out intervention programs and promote mental health.

## Data Availability

The datasets used and/or analyzed during the current study are available from the corresponding author on reasonable request.
